# Amplitude spectral area of ventricular fibrillation can discriminate survival of patients with out-of-hospital cardiac arrest

**DOI:** 10.3389/fcvm.2024.1336291

**Published:** 2024-02-06

**Authors:** Francesca Romana Gentile, Lars Wik, Iraia Isasi, Enrico Baldi, Elisabete Aramendi, Jon Erik Steen-Hansen, Alessandro Fasolino, Sara Compagnoni, Enrico Contri, Alessandra Palo, Roberto Primi, Sara Bendotti, Alessia Currao, Federico Quilico, Luca Vicini Scajola, Clara Lopiano, Simone Savastano

**Affiliations:** ^1^ Division of Cardiology, Fondazione IRCCS Policlinico San Matteo, Pavia, Italy; ^2^ Department of Molecular Medicine, Section of Cardiology, University of Pavia, Pavia, Italy; ^3^ Oslo University Hospital, Division of Prehospital Emergency Medicine, National Service of Competence for Prehospital Acute Medicine (NAKOS), Ullevål Hospital, Oslo, Norway; ^4^ Prehospital Clinic, Doctor Car, Oslo University Hospital HF, Ullevål Hospital, Oslo, Norway; ^5^ BioRes Group, University of the Basque Country, Bilbao, Spain; ^6^ Division of Prehospital Care, Vestfold Hospital Trust, Tønsberg, Norway; ^7^ AAT 118 Pavia, Agenzia Regionale Urgenza Emergenza at Fondazione IRCCS Policlinico San Matteo, Pavia, Italy

**Keywords:** cardiac arrest, AMSA, ventricular fibrillation, defibrillation, survival, OHCA

## Abstract

**Background:**

Evidence of the association between AMplitude Spectral Area (AMSA) of ventricular fibrillation and outcome after out-of-hospital cardiac arrest (OHCA) is limited to short-term follow-up. In this study, we assess whether AMSA can stratify the risk of death or poor neurological outcome at 30 days and 1 year after OHCA in patients with an initial shockable rhythm or with an initial non-shockable rhythm converted to a shockable one.

**Methods:**

This is a multicentre retrospective study of prospectively collected data in two European Utstein-based OHCA registries. We included all cases of OHCAs with at least one manual defibrillation. AMSA values were calculated after data extraction from the monitors/defibrillators used in the field by using a 2-s pre-shock electrocardiogram interval. The first detected AMSA value, the maximum value, the average value, and the minimum value were computed, and their outcome prediction accuracy was compared. Multivariable Cox regression models were run for both 30-day and 1-year deaths or poor neurological outcomes. Neurological cerebral performance category 1–2 was considered a good neurological outcome.

**Results:**

Out of the 578 patients included, 494 (85%) died and 10 (2%) had a poor neurological outcome at 30 days. All the AMSA values considered (first value, maximum, average, and minimum) were significantly higher in survivors with good neurological outcome at 30 days. The average AMSA showed the highest area under the receiver operating characteristic curve (0.778, 95% CI: 0.7–0.8, *p* < 0.001). After correction for confounders, the highest tertiles of average AMSA (T3 and T2) were significantly associated with a lower risk of death or poor neurological outcome compared with T1 both at 30 days (T2: HR 0.6, 95% CI: 0.4–0.9, *p* = 0.01; T3: HR 0.6, 95% CI: 0.4–0.9, *p* = 0.02) and at 1 year (T2: HR 0.6, 95% CI: 0.4–0.9, *p* = 0.01; T3: HR 0.6, 95% CI: 0.4–0.9, *p* = 0.01). Among survivors at 30 days, a higher AMSA was associated with a lower risk of mortality or poor neurological outcome at 1 year (T3: HR 0.03, 95% CI: 0–0.3, *p* = 0.02).

**Discussion:**

Lower AMSA values were significantly and independently associated with the risk of death or poor neurological outcome at 30 days and at 1 year in OHCA patients with either an initial shockable rhythm or a conversion rhythm from non-shockable to shockable. The average AMSA value had the strongest association with prognosis.

## 1 Introduction

Out-of-hospital cardiac arrests (OHCAs) have an incidence of cases treated by the emergency medical services (EMS) that is estimated to be up to 97 per 100,000 inhabitants per year (1, 2). The rate of survival to hospital discharge is often lower than 10% (3). An improvement of survival rates may be related to an understanding of the factors that impact resuscitation. Several aspects have been analysed by researchers and they are reported to be associated with a better prognosis. Some examples are witnessed cardiac arrest, bystander cardiopulmonary resuscitation (CPR), presenting shockable rhythm, and early defibrillation. Among patients suffering from an OHCA, a presenting shockable rhythm is notably associated with a lower risk of death compared with a non-shockable one (4–9). Some studies have documented that the conversion from a non-shockable rhythm to a shockable one is associated with a better prognosis compared with patients who remained non-shockable during the entire resuscitation effort (10, 11).

AMplitude Spectral Area (AMSA) of ventricular fibrillation (VF) is a quantitative measure of the amplitude of ventricular fibrillation, which may be measured during resuscitation. The AMSA value correlates with intracoronary pressure during resuscitation (12) and reflects the energy status of the myocardium (a concentration of adenosine triphosphate in myocardial cells) (13). Consequently, the AMSA value has been shown to predict defibrillation success and to guide defibrillation during resuscitation in terms of timing (14–19) and energy level (20). It also predicts the return of spontaneous circulation (ROSC) (16, 21) and survival to hospital discharge (22, 23). However, studies assessing the association between AMSA and the risk of death in cardiac arrest or neurological outcome are quite few (22–24) and limited both in sample size and in follow-up duration.

The aim of our study was to assess whether AMSA values were associated with the risk of death or poor neurologic outcome in patients with a shockable rhythm (either presenting or converted from non-shockable rhythm) in OHCA both at 30 days and at 1 year after the event and also to identify which of the AMSA values studied had the best power of discrimination.

## 2 Material and methods

### 2.1 Type of study and population

This is a multicentre observational study based on a retrospective analysis of prospectively collected data. All cases of OHCAs that occurred between 1 January 2015 and 31December 2020 in the province of Pavia (Italy) and between 1 January 2007 and 31 December 2018 in Vestfold county (Norway) were eligible for inclusion. We included patients who received at least one shock for VF during advanced resuscitation, regardless of whether the first rhythm was shockable. Pre-hospital and outcome data were retrieved from the respective cardiac arrest registries: the Lombardia CARe Registry and the Vestfold Cardiac Arrest Registry.

The areas of these registries included in the present study were the province of Pavia and the region of Vestfold, respectively. Both registries follow the Utstein recommendations (25) and have been approved by the respective ethic committees, and accordingly, every patient with a sufficient neurologic recovery signed an informed consent form. The two emergency systems and the two registries are described in Supplementary Appendix S1.

### 2.2 AMSA calculation

**Figure 1:**
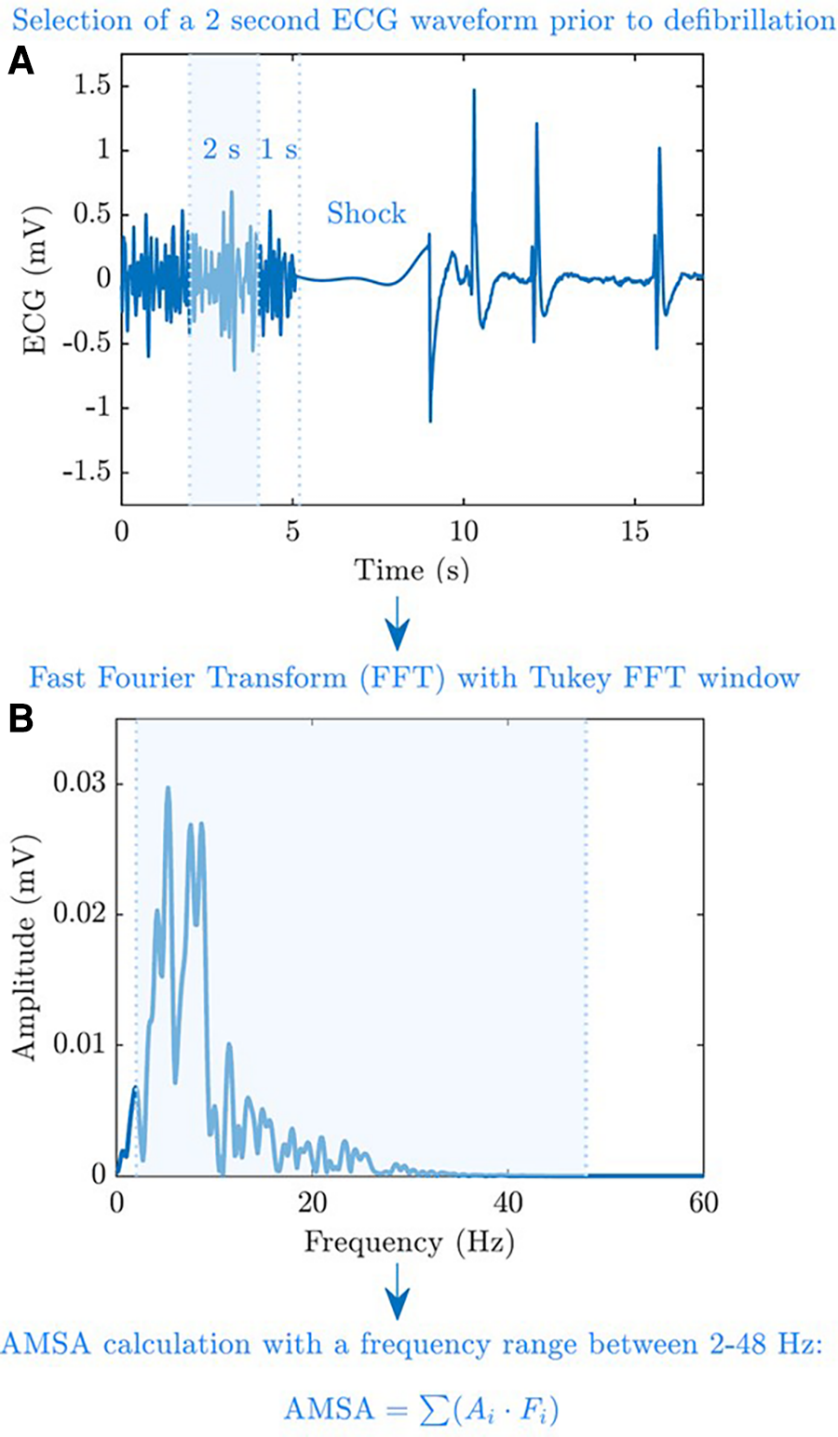
AMSA calculation process. AMSA was computed using a 2 s pre-shock ECG interval and free of chest compression artefacts, leaving a 1 s guard before the shock (**A**). Fast Fourier Transform with 1,024 points was used to compute the spectral amplitudes of the ECG, and AMSA was calculated at a 2–48 Hz frequency range (**B**).

After extracting the electronic data of each OHCA from the storage of monitor/defibrillators (Corpuls 3 for the province of Pavia and Lifepak 12/15 monitors for Vestfold region), ECG signals were processed by using Matlab software (The MathWorks, Inc., Natick, MA, USA). For each shock, AMSA was computed using a 2 s pre-shock ECG interval, free of chest compression artefacts, leaving 1 s guard before the shock (Figure 1A). The ECG waveform of Corpuls 3 was resampled to a common sampling rate of 250 Hz used by LifePak monitors. The signal was then bandpass-filtered (0.5–30 Hz) using a forward–backward order 8 elliptic filter to remove baseline oscillations and high-frequency noise and windowed with a Tukey window. Fast Fourier Transform with 1,024 points was used to compute the spectral amplitudes of the ECG, and AMSA was calculated in the 2–48 Hz frequency range as previously described (14) (Figure 1B). For each patient, the value of AMSA of the first shock delivered by the advance resuscitation team and the maximum, minimum, and average values of AMSA were annotated during resuscitation. Moreover, we considered the presence of an increase in AMSA values during resuscitation. In this regard, an increase in AMSA values was observed if the first value of AMSA recorded was lower than the maximum value obtained during advanced resuscitation.

### 2.3 Outcome definitions

We followed Utstein definitions of outcome (25). ROSC was assumed, even if transient, in the presence of a palpable pulse checked according to the guidelines (26, 27). ROSC was annotated by clinicians on scene after each shock. The survival of the event was defined as the maintenance of ROSC until hospital admission or transfer of care to medical staff. Survival at 30 days was a core outcome of both registries. Survival at 1 year was available only for patients enrolled in the Lombardia CARe registry.

Neurological outcome was graded using the cerebral performance category (CPC) scale, which is divided into five classes described as follows. CPC 1: full neurological recovery or mild disability; CPC 2: moderate disability but independent in daily living activities; CPC 3: severe disability and dependent in daily living activities; CPC 4: persistent vegetative status; CPC 5: dead. CPC 1 or 2 was defined as good neurological outcome (25).

### 2.4 Statistical analysis

Data from the two different databases were integrated and combined in a single *ad hoc* database for statistical analysis. Categorical variables were compared by using the *χ*^2^ test and presented as numbers and percentages. Continuous variables were compared by using the *t*-test and presented as mean ± standard deviation or by using the Mann–Whitney test and presented as median (interquartile range, IQR) according to normal distributions (examined with the D’Agostino–Pearson test). The degree of relationship between the variables can be determined using rank correlation, and Spearman's ρ was considered. The outcome prediction accuracy of AMSA was tested using the receiver operating characteristic (ROC) curve analysis; the area under the curve (AUC) was calculated according to the Hanley and McNeil methodology. A comparison of the ROC curves was performed according to the DeLong method. Uni- and multivariable Cox regression models were run to test the effect of one or more non-correlated variables on the risk of death or poor neurological outcome.

We inserted in the multivariable models only a non-correlated variable with a *p*-value <0.1 in the univariable model. Statistical analyses were conducted by using the software MedCalc version 20.210. A *p*-value <0.05 was considered statistically significant.

## 3 Results

### 3.1 Study population characteristics

A total of 629 patients with OHCAs were enrolled in the study and outcome data were available for 578 patients: 236 from Pavia and 342 from Vestfold. Out of these, 91% had a medical aetiology, 68% occurred at home, and 77% were males; the median age was 68 (IQR, 57–77) years old. The rate of bystander CPR was 73%, 63% had a shockable presenting rhythm, and 13% (74) of the patients were alive with a good neurological outcome at 30 days. Those who survived were younger, with a shorter call to EMS arrival time, more frequently had a shockable presenting rhythm, and less frequently resuscitated at home. The other characteristics are presented in Table 1.

**Table 1:** Patient characteristics based on their survival status and neurological outcome.

Variable	Overall	Alive with CPC ≤ 2 at 30 days	Dead or CPC > 2 at 30 days	*p*-value
	*N* = 578	*N* = 74	*N* = 504	
Study site (%)				0.29
Pavia	236 (41)	26 (35)	210 (42)	
Vestfold	342 (59)	48 (65)	294 (58)	
Age (IQR) (years)	68 (57–77)	63 (52–72)	69 (59–78)	<0.001
Male gender (%)	450 (77)	58 (78)	392 (78)	0.95
EMS arrival time (IQR) (min)	9.5 (6.9–13.4)	8.2 (6–11.7)	9.9 (7.1–13.9)	0.006
Medical aetiology (%)	525 (91)	71 (96)	454 (90)	0.07
Home location (%)	394 (68)	34 (46)	360 (71)	<0.001
Telephone CPR (%)	293 (51)	36 (48)	257 (51)	0.78
Witnessed event				<0.001
EMS (%)	58 (10)	20 (27)	38 (8)	
Bystanders (%)	398 (69)	48 (65)	350 (69)	
Bystander CPR (%)^a^	382 (73)	44 (81)	338 (73)	0.11
Shockable presenting rhythm (%)	362 (63)	68 (91)	294 (58)	<0.001
Epinephrine (IQR) (mg)	5 (3–6)	1 (0–2)	5 (3–6)	<0.001
Rhythm conversion from non-shockable to shockable (%)	216 (37)	6 (9)	210 (42)	<0.001
AED use before EMS arrival (%)^a^	70 (13)	7 (13)	63 (13)	0.52
Number of shocks delivered (IQR)	3 (1–6)	2 (1–4)	3 (1–6)	0.13
Mechanical CPR (%)	364 (63)	27 (36)	337 (66)	<0.001

AED, Automated External Defibrillator.

^a^ EMS witnessed excluded.

### 3.2 AMSA and short-term survival with good neurological outcome

**Figure 2:**
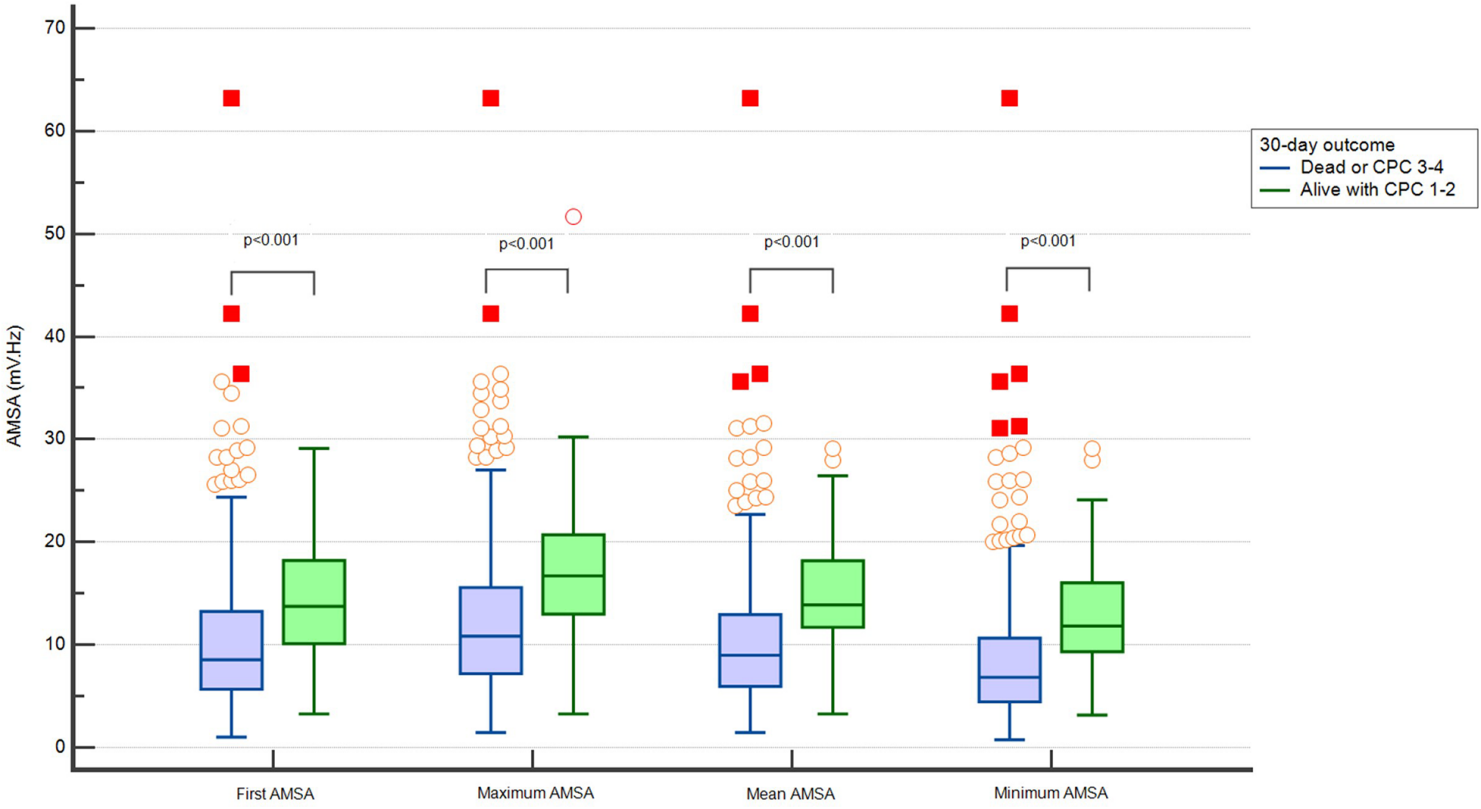
Distribution of different values of AMSA in patients dead or with CPC > 2 and alive with CPC ≤ 2 at 30 days.

The first detected AMSA value and the maximum, average, and minimum values of AMSA were higher in patients who survived with good neurological outcome at 30 days (Figure 2).

The maximum, minimum, and average AMSA values correlated with the first AMSA value (ρ 0.81, 0.85, and 0.88, respectively, *p* < 0.001) (Supplementary Figure 1S). All analysed AMSA values showed a good discriminatory performance predicting survival and the average AMSA had the highest value of area under the ROC curve [0.778 (0.74–0.81)] (Supplementary Table 1S).

The whole study population was divided into three tertiles according to the average AMSA values: T1 1.4–7.7 mV Hz; T2 7.7–12.7 mV Hz; T3 12.7–63.1 mV Hz. The characteristics of patients with a higher AMSA value were a lower EMS arrival time, lower number of shocks, higher rate of witnessed event, and shockable presenting rhythm. The other characteristics are presented in Table 2.

**Table 2:** Patient characteristics according to the average AMSA tertiles.

Variable	Average AMSA			*p*-value
	T1	T2	T3	
	1.4–7.7 mV Hz	7.7–12.7 mV Hz	12.7–63.1 mV Hz	
Age (IQR) (years)	69 (58–78)	67 (57–76)	68 (56–77)	0.6
Number of shocks (IQR)	3 (1–7)	4 (1–6.5)	2 (1–5)	<0.001
Epinephrine (mg) (IQR)	5 (4–7)	5 (2–6)	3 (2–5)	<0.001
EMS arrival time	10 (7.4–14.2)	10 (6.9–13.9)	8.8 (6.8–12.3)	0.01
Male gender (%)	153 (81)	148 (84)	123 (74)	0.06
Home location (%)	135 (73)	125 (71)	103 (62)	0.12
Medical aetiology (%)	173 (91)	168 (95)	152 (92)	0.24
Witnessed event (%)	139 (73)	146 (84)	146 (90)	<0.001
Bystanders CPR (%)	131 (69)	111 (65)	117 (72)	0.35
Telephone CPR (%)	94 (50)	84 (50)	93 (57)	0.31
Shockable presenting rhythm (%)	107 (56)	130 (68)	125 (65)	<0.001
Rhythm conversion from non-shockable to shockable (%)	83 (44)	65 (32)	68 (35)	<0.001
Mechanical CPR (%)	128 (68)	111 (64)	94 (57)	0.04

**Figure 3:**
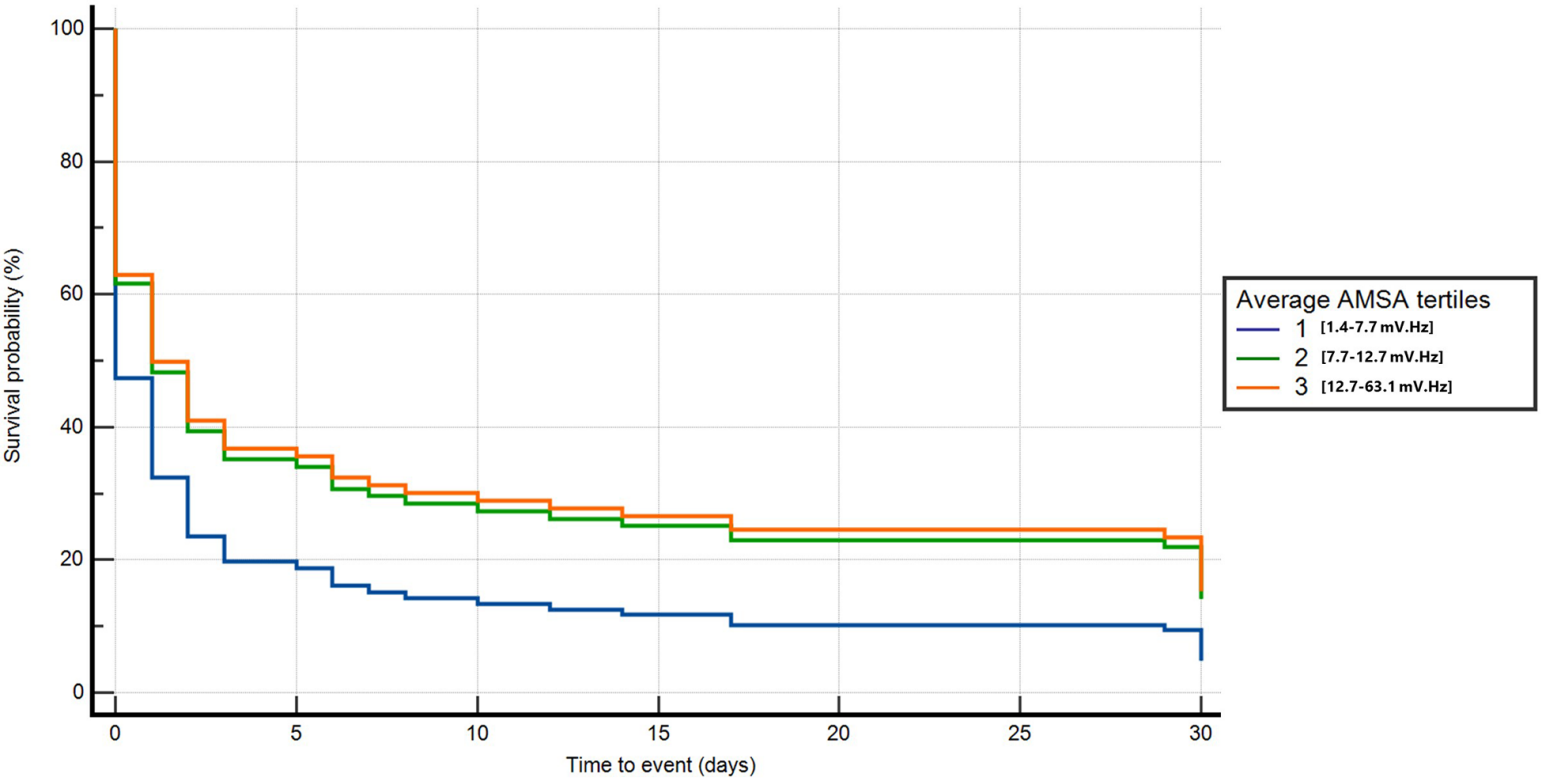
Multivariable Cox regression for death or poor neurological outcome at 30 days neurological outcome.

In the univariable Cox regression analysis, T2 and T3 were significantly associated with a lower probability of death or poor neurological outcome compared with T1 (T2: HR 0.7, 95% CI: 0.5–0.8, *p* < 0.001; T3: HR 0.5, 95% CI: 0.4–0.6, *p* < 0.001). In the multivariable analysis, after adjusting for age, home location, witnessed status, study site, the use of mechanical CPR, EMS arrival time, the amount of epinephrine administered, and conversion from non-shockable to shockable rhythm, T2 and T3 were confirmed to be independently and significantly associated with a lower risk of death or poor neurological outcome compared with T1 (T2: HR 0.6, 95% CI: 0.4–0.9, *p* = 0.01; T3: HR 0.6, 95% CI: 0.4–0.9, *p* = 0.02) (Figure 3 and Supplementary Appendix S2).

### 3.3 The increase in AMSA during resuscitation and 30-day survival

**Figure 4:**
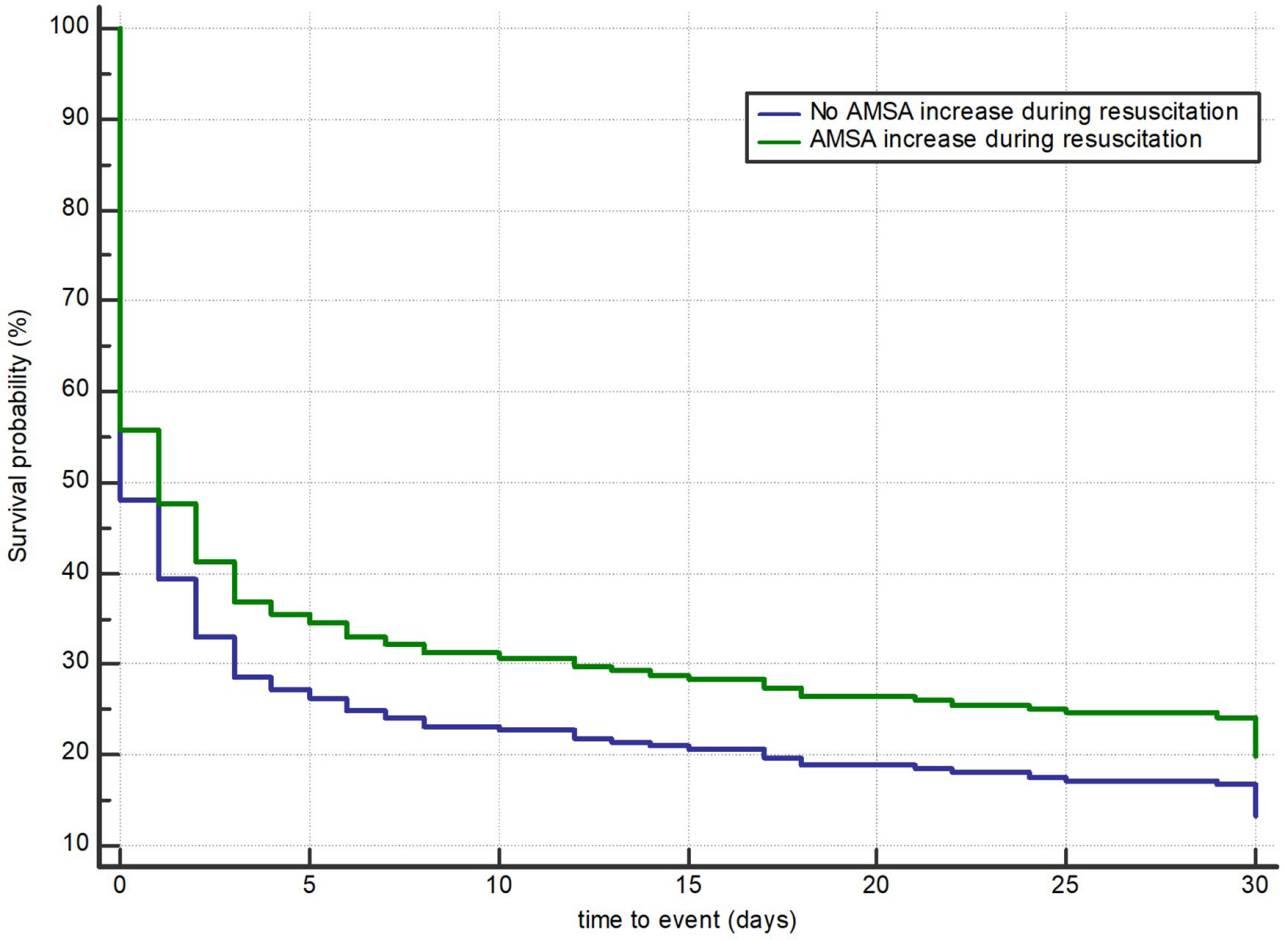
Multivariable Cox regression model for death or poor neurological outcome according to an observable increase in AMSA values.

In 254 (44%) of the 578 patients, AMSA values increased during resuscitation. These patients, compared with those without an increase in AMSA, showed a higher rate of male gender, medical aetiology of cardiac arrest, witnessed event, bystander CPR, shockable rhythm, a higher number of shocks delivered, and a higher rate of 30-day survival with good neurological outcome (16% vs. 10%, *p* = 0.03) (Table 3). In the multivariable Cox regression analysis, the increase in AMSA values was found to be significantly associated with the risk of death or poor neurological outcome at 30 days after correction for the first AMSA value measured, age, gender, study site, number of shocks received, OHCA location, aetiology, witnessed status, presence of a bystander, telephone CPR, EMS arrival time, use of amiodarone, mechanical CPR, and conversion from non-shockable to shockable rhythm (HR 0.8, 95% CI: 0.63–0.99, *p* = 0.047) (Figure 4 and Supplementary Appendix S2).

**Table 3:** Patient characteristics according to an observable increase in AMSA during resuscitation.

Variable	No AMSA increase	AMSA increase	*p*-value
	*n* = 324	*n* = 254	
Age (IQR) (years)	68.5 (58–78)	68 (55–76)	0.24
Number of shock (IQR)	1 (1–3)	5 (3–7)	<0.001
Epinephrine (IQR) (mg)	5 (3–6)	4.5 (3–6)	0.88
EMS arrival time	9.5 (6.9–13.8)	9.3 (6.9–13)	0.44
Male gender (%)	231 (71)	249 (98)	<0.001
Home location (%)	223 (69)	191 (75)	0.17
Medical aetiology (%)	306 (94)	254 (100)	0.01
Witnessed event (%)	222 (68)	203 (80)	0.01
Bystanders CPR (%)	217 (67)	197 (77)	0.03
Telephone CPR (%)	161 (50)	155 (61)	0.02
Shockable presenting rhythm (%)	175 (54)	197 (77)	<0.001
Rhythm conversion from non-shockable to shockable (%)	149 (46)	57 (23)	<0.001
Mechanical CPR (%)	213 (66)	176 (69)	0.42
30-day survival with CPC ≤ 2	33 (10)	41 (16)	0.03

### 3.4 AMSA and long-term survival with good neurological outcome

One-year survival status was available for the 236 patients from Pavia; of these, 22 were alive at 1 year with a CPC ≤2.

**Figure 5:**
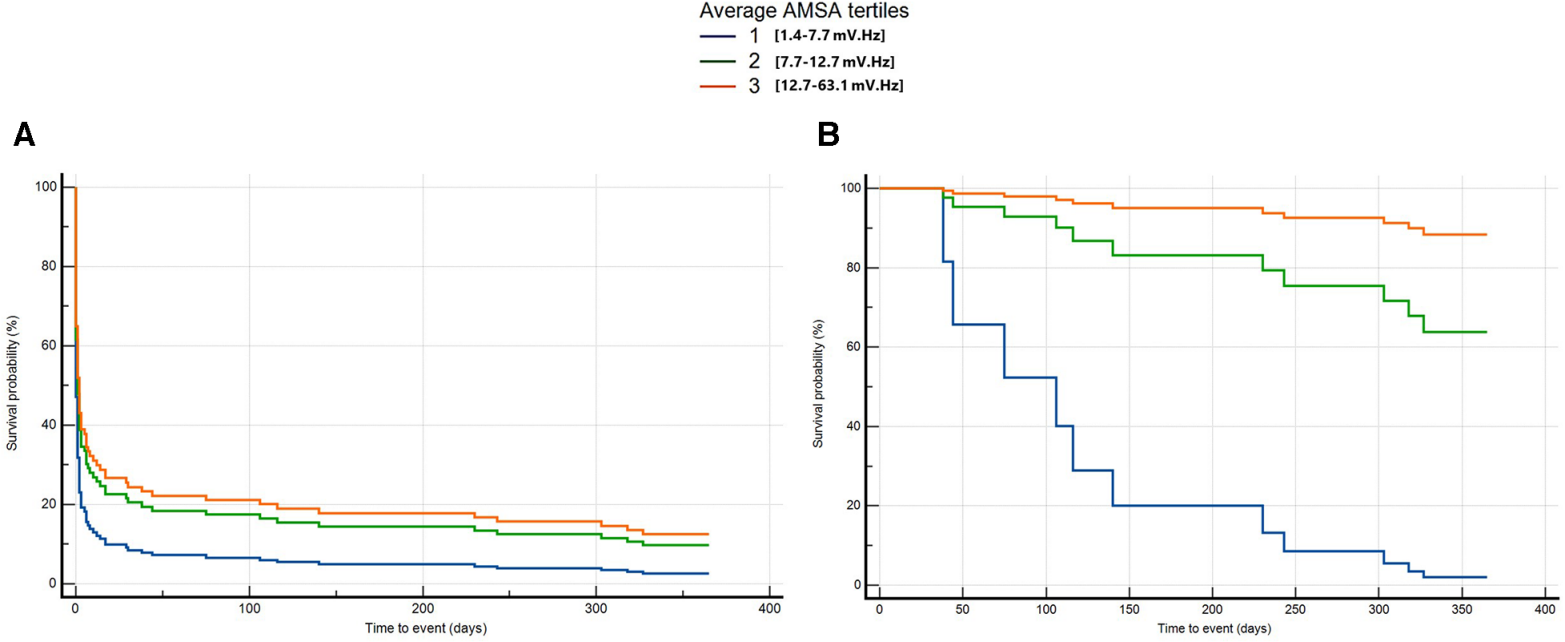
Multivariable Cox regression for long-term death or poor neurological outcome considering all patients from OHCA (**A**) and only patients alive at 30 days (**B**).

The average T2 and T3 was significantly associated with the probability of death or poor neurological outcome at 1 year compared with T1 after adjusting for age, home location, witnessed status, the use of mechanical CPR, EMS arrival time, the amount of epinephrine, and conversion from non-shockable to shockable rhythm (T2: HR 0.6, 95% CI: 0.4–0.9, *p* = 0.01; T3: HR 0.6, 95% CI: 0.4–0.9, *p* = 0.01) (Figure 5A and Supplementary Appendix S2). By considering those patients who survived at 30 days, we found that those with a higher AMSA had a lower risk of death or poor neurological outcome at 1 year after OHCA in the univariable Cox regression analysis (T3 vs. T1: HR 0.04, 95% CI: 0–0.6, *p* = 0.02). After correcting for age, EMS arrival time, and the amount of epinephrine administered, T3 was confirmed to be independently and significantly associated with a lower risk of death or poor neurological outcome compared with T1 at 1 year after OHCA (T3: HR 0.03, 95% CI: 0–0.3, *p* = 0.02) (Figure 5B and Supplementary Appendix S2).

## 4 Discussion

The main findings of this study are as follows: (1) all the values of AMSA analysed during resuscitation (the first value measured, the maximum value, the minimum value, and the average AMSA) were significantly associated with the risk of death or poor neurological outcome at 30 days; (2) the average AMSA had the best discriminatory power in predicting survival among all; (3) the average AMSA, even when adjusted for cofounders, was associated with short-term survival (at 30 days) with good neurological outcome; (4) the increase in AMSA values during resuscitation was associated with short-term survival with good neurological outcome, (5) the average AMSA, adjusted for confounders, was also associated with long-term outcome (1 year) after OHCA.

The positive association between AMSA values and survival in OHCA was proposed about 10 years ago (23). Indik et al., indeed, found that the average AMSA was associated with a good neurological outcome at discharge from a sample of 89 OHCA patients (23), and subsequently, they confirmed this result on a larger sample of 140 patients (24). We are in agreement with their conclusion because the area under the ROC curve that they reported is similar to the ones we demonstrated in this paper, and the values of the average AMSA associated with a good neurological outcome correspond to our third tertile of average AMSA values. On a sample of 390 OHCA patients, Schoene et al. (22) showed that the values of AMSA prior to each shock, as well as the variation of AMSA values, were associated with favourable neurologic survival at discharge. Compared with the aforementioned studies, we present the first multicentre study that has proved that a higher AMSA value is significantly and independently associated with a better outcome at 30 days and at 1 year after OHCA. Our study was based on a larger sample from two different emergency medical services and OHCA registries. As previously suggested, we have also confirmed that the increase in AMSA values during resuscitation is associated with a better prognosis at 30 days. This may be the result of good quality resuscitation, able to restore the myocardial energetic supplies.

In our study, patients with an initial shockable rhythm, but in the lowest AMSA tertile, had similar outcomes to those with a non-shockable rhythm despite being shockable. This demonstrates that the amplitude of the VF underlies a prognostic role that may exceed the standard distinction between shockable and non-shockable rhythms used by the Utstein recommendations.

One interesting point that distinguishes our study from the others is the inclusion criteria. In contrast to previous studies (22–24), which enrolled patients only with a shockable presenting rhythm, we decided to consider all OHCAs with at least one shock administered during advanced resuscitation. Therefore, we included patients with an initial non-shockable rhythm, which then converted into a shockable rhythm during resuscitation. In our population, this category represented 37% of all the OHCAs. Generally, the conversion from a non-shockable rhythm to a shockable rhythm correlates with a better prognosis compared with a persistent non-shockable rhythm (11). However, the prognostic role of rhythm conversion is controversial, probably because not all the shockable rhythms are the same. In this category of patients, the amplitude of the shockable rhythm that occurs after the conversion may be more important than rhythm conversion itself, as outlined by our results. However, larger studies focusing on this aspect are needed to confirm this hypothesis.

For the first time, we provided a direct comparison between the different values of AMSA measured, and we identified that the average AMSA was the one with the best discriminatory performance in predicting survival. In contrast, Indik et al. tested only one value of AMSA, whereas Schoene et al. tested separately different values of AMSA and their variations, without providing a direct comparison between them (22).

We showed that a higher AMSA value was associated with a better survival with good neurological outcome at 1 year after cardiac arrest. Notably, we provided two different analyses for long-term outcomes: one focused on 1-year survival of all the OHCA patients and one focused on 1-year survival of patients alive at 30 days. By doing so, we support the hypothesis of an additional risk of death after 30 days according to the average AMSA values recorded during the resuscitation effort.

The reasons why AMSA may be associated with survival are probably multiple, but the essential core is that AMSA reflects the metabolic status of the myocardium. Higher AMSA values are the result of a higher energy store in myocardial cells from which it derives a higher chance of shock success. This can support a faster recovery and a lower rate of in-hospital death. Of course, the metabolic state of the heart depends on other unquestionable characteristics of the event. It is not surprising that patients in the lower tertile of AMSA had more frequently a non-shockable presenting rhythm, an unwitnessed event at home, and received more epinephrine. These characteristics are typically present in many scoring systems such as the cardiac arrest hospital prognosis (CAHP) score (28). However, these scoring systems often include variables that are available only after hospital admission. Instead, AMSA, which reflects the effect of all these variables on the heart during resuscitation, may be available on the field allowing an earlier prognostication in cardiac arrest.

Explaining why AMSA could be associated with a long-term outcome is indeed a challenging task. Because previous studies showed that AMSA values were generally low in patients with acute or chronic myocardial infarction (21, 29, 30) or with increased left ventricle diameter (31), it is possible that the patients in the lower tertile of the average AMSA are more likely to have structural heart disease and consequently a worse long-term outcome.

On the basis of the recent data supporting the feasibility of real-time AMSA measurement during resuscitation (32), of the useful role of AMSA during resuscitation, as a predictor of shock success and ROSC, and on the basis of the results of the present study, we hypothesise that AMSA should be available in the field. Defibrillators companies should make efforts in this direction to help rescuers to tailor both resuscitation and post-ROSC care.

### 4.1 Limitations

The first limitation is that this an observational study based on registries and not a prospective study. The second limitation is that AMSA was calculated *post hoc* instead of being real-time annotated during resuscitation. Prospective studies with AMSA measured directly on the field are needed to confirm our results. The third limitation is that long-term outcome was available only in a subgroup of patients. This is because the Utstein template limits the follow-up up to 30 days, while longer periods are only optional for registries based on the Utstein template. However, a total of 236 patients represent a good sample that is bigger than those considered in previous studies; despite this fact, larger studies are needed to confirm our results.

## 5 Conclusion

This study demonstrates that AMSA values are associated with both short- and long-term survival in OHCA patients with a shockable rhythm and that the average AMSA is the value with the best discriminatory power among all others. If confirmed in a prospective study, our results could help tailor both resuscitation and post-ROSC care.

## Data Availability

The raw data supporting the conclusions of this article will be made available by the authors, under specific request to the corresponding author.

## Supplementary material

Supplementary Appendix S1 Here are described the two emergency systems and the two OHCA registries.

Supplementary Appendix S2 Here are described the multivariable Cox regression model results.

Supplementary Figure S1 Correlation between the first AMSA value and maximum, average and minimum AMSA values.
