# Supplementary material for: Amplitude spectral area of ventricular fibrillation can discriminate survival of patients with out-of-hospital cardiac arrest
Source: Front Cardiovasc Med. 2024 Feb 6;11:1336291. doi: 10.3389/fcvm.2024.1336291 (PMC10876863; doi:10.3389/fcvm.2024.1336291)
Supplement: Supplementary file 1 [file Table1.docx]

**Table 1S. Comparison of ROC curves**

|  | AUC | 95% CI | p value | p value vs First AMSA |
| --- | --- | --- | --- | --- |
| First AMSA | 0.7 | 0.70-0.77 | <0.001 | - |
| Maximum AMSA | 0.75 | 0.71-0.78 | <0.001 | 0.57 |
| Minimum AMSA | 0.77 | 0.73-0.80 | <0.001 | 0.07 |
| Average AMSA | **0.78** | 0.74-0.81 | <0.001 | **0.007** |
