# Supplementary material for: Amplitude spectral area of ventricular fibrillation can discriminate survival of patients with out-of-hospital cardiac arrest
Source: Front Cardiovasc Med. 2024 Feb 6;11:1336291. doi: 10.3389/fcvm.2024.1336291 (PMC10876863; doi:10.3389/fcvm.2024.1336291)
Supplement: Supplementary Appendix S2 — Here are described the multivariable Cox regression model results. [file Datasheet2.docx]

**Supplementary appendix 2**

**Multivariable Cox regression for 30-day mortality or poor neurological outcome**

| **Covariate** | **HR** | **95% CI** | **P value** |
| --- | --- | --- | --- |
| **Average AMSA T1** | Ref |  |  |
| **Average AMSA T2** | 0.6453 | 0.4526 - 0.9200 | 0.0155 |
| **Average AMSA T3** | 0.6173 | 0.4089 - 0.9320 | 0.0217 |
| **Age (year)** | 1.0117 | 0.9998 - 1.0236 | 0.0534 |
| **Witnessed event** | 0.7 | 0.4549 - 1.0121 | 0.0573 |
| **Adrenaline (mg)** | 1.1164 | 1.0583 - 1.1776 | 0.0001 |
| **Manual CPR** | 0.8528 | 0.6199 - 1.1731 | 0.3277 |
| **EMS arrival time (min)** | 1.0093 | 0.9748 - 1.0451 | 0.6008 |
| **Home location** | 0.9193 | 0.6537 - 1.2930 | 0.6288 |
| **Rhythm conversion** | 1.4160 | 1.0243 - 1.9576 | 0.0353 |

**Multivariable Cox regression for 30-day mortality or poor neurological outcome**

| **Covariate** | **HR** | **95% CI** | **p value** |
| --- | --- | --- | --- |
| **AMSA increase** | 0.8 | 0.6-0.99 | 0.0471 |
| **First AMSA** | 0.99 | 0.96-0-99 | 0.0120 |
| **Male sex** | 1.04 | 0.81-1.33 | 0.7696 |
| **Study site** | 0.93 | 0.75-1.15 | 0.4979 |
| **Number of shocks** | 1.04 | 1.005-1.07 | 0.0238 |
| **Amiodarone** | 1 | 0.8-1.3 | 0.9810 |
| **Home location** | 1.2 | 0.9-1.5 | 0.1747 |
| **Bystander CPR** | 1.1 | 0.8-1.5 | 0.4202 |
| **Witnessed event** | 0.8 | 0.6-1.1 | 0.2158 |
| **Age (years)** | 1.01 | 1.003-1.02 | 0.0073 |
| **EMS arrival time (min)** | 1.01 | 0.994-1.023 | 0.2272 |
| **Rhythm conversion** | 0.7 | 0.5-0.8 | 0.0009 |
| **Use of mechanical CPR** | 1.3 | 1.04-1.6 | 0.0198 |
| **Telephone CPR** | 1 | 0.8-1.3 | 0.9615 |
| **Medical aetiology** | 0.9 | 0.6-1.3 | 0.5073 |

**Multivariable Cox regression for one-year mortality or poor neurological outcome**

| **Covariate** | **HR** | **95% CI** | **P value** |
| --- | --- | --- | --- |
| **Average AMSA T1** | Ref |  |  |
| **Average AMSA T2** | 0.6432 | 0.4508 - 0.9176 | 0.0149 |
| **Average AMSA T3** | 0.5725 | 0.3808 - 0.8606 | 0.0073 |
| **EMS arrival time (min)** | 1.0073 | 0.9733 - 1.0424 | 0.6784 |
| **Age (year)** | 1.0113 | 1.0001 - 1.0227 | 0.0474 |
| **Unwitnessed event** | 1.4553 | 0.9702 - 2.1828 | 0.0697 |
| **Manual CPR** | 1.0058 | 0.9861 - 1.0258 | 0.5669 |
| **Home location** | 1.0849 | 0.7731 - 1.5222 | 0.6374 |
| **Non-shockable presenting rhythm** | 1.4164 | 1.0248 - 1.9577 | 0.0350 |
| **Adrenaline (mg)** | 1.1257 | 1.0694 - 1.1850 | <0.0001 |

**Baseline cumulative hazard function** [[Show]](javascript:showdiv('d56','d57','table1');)

**Multivariable Cox regression for one-year mortality or poor neurological outcome considering patients alive at 30 days**

| **Covariate** | **HR** | **95% CI** | **P** |
| --- | --- | --- | --- |
| **Average AMSA T1** | ref |  |  |
| **Average AMSA T2** | 0.1149 | 0.0079 - 1.6624 | 0.1125 |
| **Average AMSA T3** | 0.0316 | 0.0016 - 0.6397 | 0.0244 |
| **EMS Arrival time (min)** | 1.1282 | 0.9334 - 1.3637 | 0.2122 |
| **Age (year)** | 1.0525 | 0.9829 - 1.1269 | 0.1425 |
| **Adrenaline (mg)** | 1.3274 | 0.9633 - 1.8291 | 0.0834 |

**Baseline cumulative hazard function** [[Show]](javascript:showdiv('d60','d61','table1');)
