# Supplementary figures and images for: Amplitude spectral area of ventricular fibrillation can discriminate survival of patients with out-of-hospital cardiac arrest
Source: Front Cardiovasc Med. 2024 Feb 6;11:1336291. doi: 10.3389/fcvm.2024.1336291 (PMC10876863; doi:10.3389/fcvm.2024.1336291)

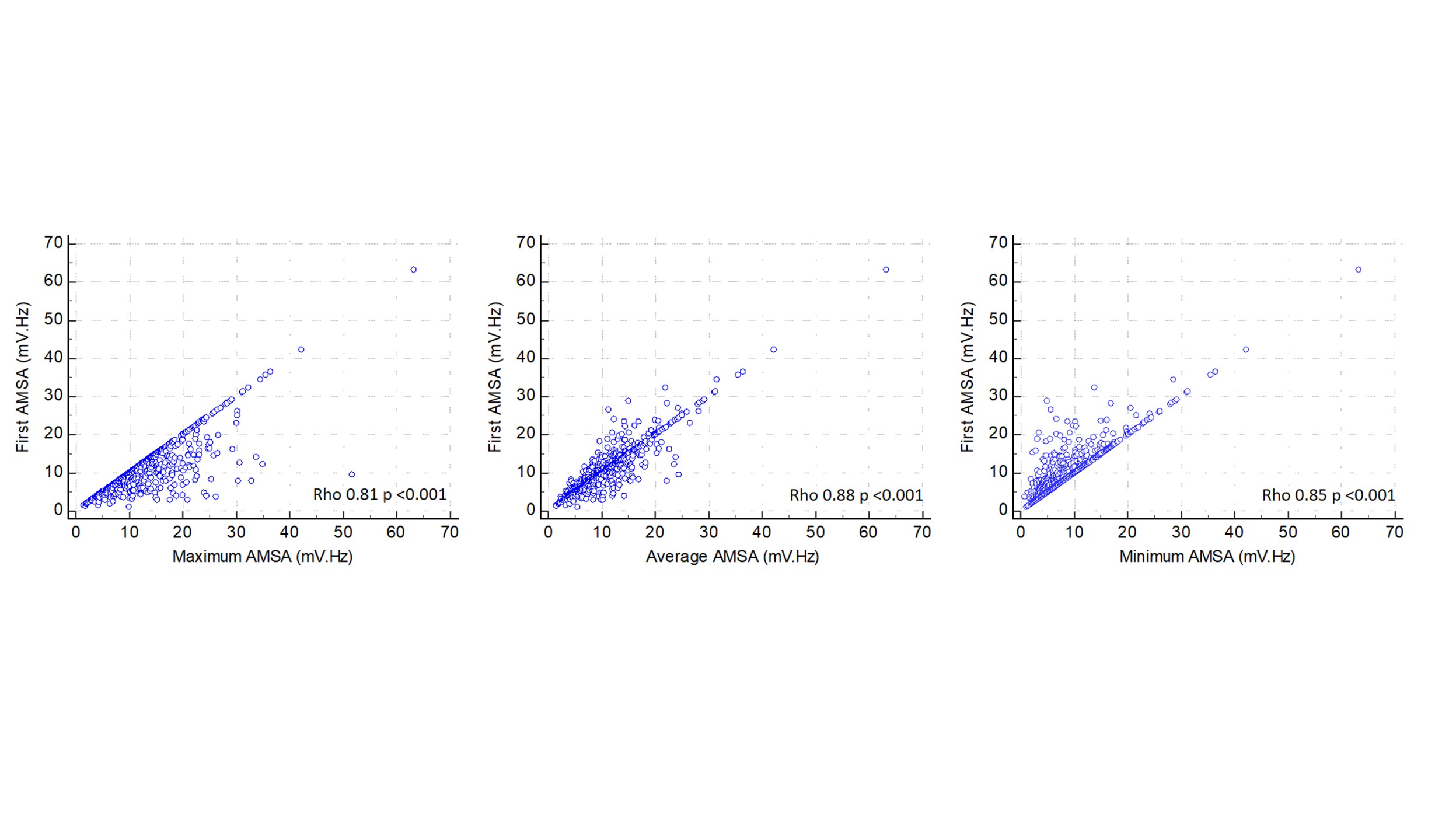

Supplement: Supplementary Figure S1 — Correlation between the first AMSA value and maximum, average and minimum AMSA values. [file Image1.jpeg]
